# Novel Anterior Brainstem Magnetic Resonance Imaging Findings in Non-Small Cell Lung Cancer with Leptomeningeal Carcinomatosis

**DOI:** 10.3389/fneur.2017.00579

**Published:** 2017-10-31

**Authors:** Chun-Yu Cheng, Chia-Yu Hsu, Yuan-Hsiung Tsai, Kuang-Lin Lin, Cih-En Huang, Yi-Hong Fan, Shy-Chyi Chin, Yen-Chu Huang

**Affiliations:** ^1^Department of Neurosurgery, Chang Gung Memorial Hospital, Chiayi, Taiwan; ^2^College of Medicine, Chang Gung University, Taoyuan, Taiwan; ^3^Department of Neurology, Chang Gung Memorial Hospital, Chiayi, Taiwan; ^4^Department of Diagnostic Radiology, Chang Gung Memorial Hospital, Chiayi, Taiwan; ^5^Department of Pediatric Neurology, Chang Gung Children’s Hospital, Taoyuan, Taiwan; ^6^Division of Hematology and Oncology, Department of Internal Medicine, Chang Gung Memorial Hospital, Chiayi, Taiwan; ^7^Division of Chest, Department of Internal Medicine, Chang Gung Memorial Hospital, Chiayi, Taiwan; ^8^Department of Diagnostic Radiology, Chang Gung Memorial Hospital, Taoyuan, Taiwan

**Keywords:** brainstem, restrictive diffusion, brain magnetic resonance imaging, leptomeningeal carcinomatosis, lung cancer

## Abstract

Leptomeningeal carcinomatosis (LC) is found in around 4% of patients with non-small cell lung cancer (NSCLC). The most common radiological finding of LC is diffuse leptomeningeal enhancement on contrast-enhanced brain magnetic resonance imaging (MRI). Herein, we report a novel brain MRI finding—non-enhanced, band-like, symmetric restricted diffusion along the anterior surface of the brainstem—of LC in four patients with NSCLC. We also identified three additional cases with similar MRI findings in a literature review. We hypothesized that the restricted diffusion along the anterior brainstem was caused by malignant cells concentrating in the cistern around the brainstem and infiltrating into the circumferential perforating arteries along the anterior brainstem surface, which then resulted in microinfarctions.

## Introduction

Leptomeningeal carcinomatosis (LC) is found in around 4% of patients with non-small cell lung cancer (NSCLC), and it is associated with a median survival of only 5 months (1). Although diffuse leptomeningeal enhancement in contrast-enhanced brain magnetic resonance imaging (MRI) is the most sensitive and non-invasive diagnostic tool for LC, false-negative, or non-specific brain MRI findings have been reported in 30% of patients (2). Herein, we report a novel brain MRI finding in four patients with NSCLC and LC, and discuss the findings of a literature review.

## An Illustrative Case

**Figure 1:**
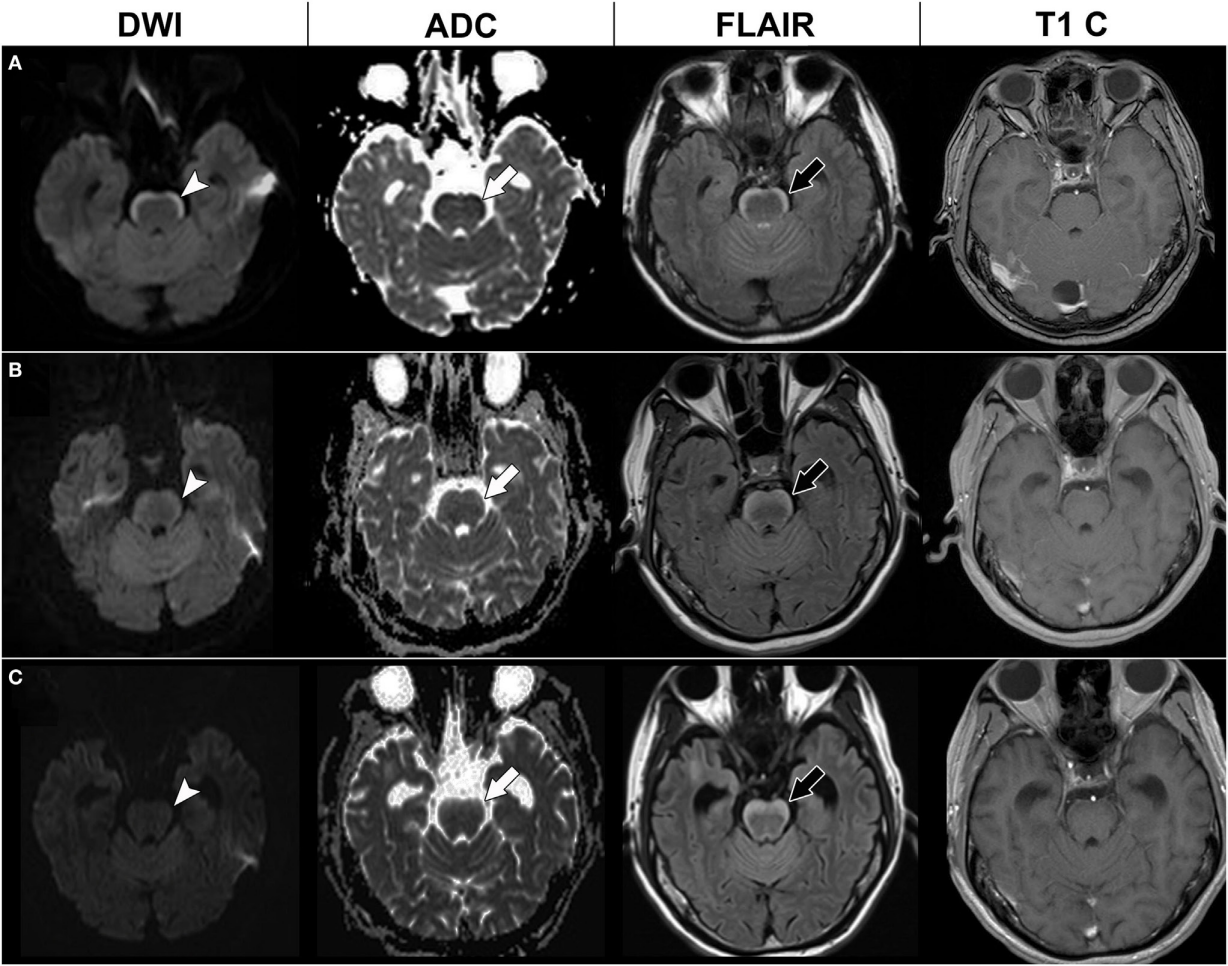
Serial brain magnetic resonance imaging (MRI) of case 1. Initial brain MRI **(A)** showed a high signal in diffusion-weighted imaging (DWI) at the anterior pons (arrow head) with a low signal in apparent diffusion coefficient (ADC) mapping (white arrow), hyperintensities in fluid attenuation inversion recovery (FLAIR) imaging (black arrow), and no enhancement in T1-weighted imaging with contrast (T1 C). Brain MRI 4 months later **(B)** and 7 months later **(C)** showed a thickening of the anterior pons lesion in the FLAIR imaging (black arrow), but gradual resolution of the restricted diffusion pattern in DWI (arrow head) and ADC mapping (white arrow).

A 39-year-old male smoker with diabetes mellitus was diagnosed with lung adenocarcinoma with epidermal growth factor receptor (EGFR) gene mutations in June 2011. He was treated with tumor resection and adjuvant chemotherapy (vinorelbine and cisplatin). Lung-to-lung metastasis developed in March 2012, after which he was treated with gefitinib. In July 2012, he presented with progressive dizziness, tinnitus, hearing impairment, seizures, and unsteady gait. A neurological examination revealed nystagmus, binocular diplopia, and ataxic gait. Brain MRI showed a non-enhanced band-like lesion along the anterior surface of the middle cerebellar peduncle, pons, and medulla with a high signal on fluid attenuation inversion recovery (FLAIR) imaging, and a restricted diffusion pattern on diffusion-weighted imaging (DWI) and apparent diffusion coefficient (ADC) mapping (Figure 1). Cerebrospinal fluid (CSF) analysis showed mild pleocytosis (white blood cell count 7/mm^3^), elevated protein (376.9 mg/dl), elevated carcinoembryonic antigen (805.9 ng/dl), and a negative oligoclonal band. CSF cytology showed malignant cells. Serum anti-Ma2 antibodies and anti-glutamic acid decarboxylase antibodies were positive.

Our initial diagnosis was paraneoplastic brainstem encephalitis. Intravenous steroids and intravenous immunoglobulin were given in September 2012; however, his dizziness and ataxic gait did not improve. Under the further suspicion of LC, intrathecal chemotherapy with cytarabine and methotrexate was administered, with gefitinib being shifted to erlotinib in November 2012. Follow-up brain MRI after 3 months showed persistent hyperintensity on FLAIR imaging but regression of restricted diffusion of the anterior brainstem lesions (Figure 1). However, he experienced progressive general weakness in the following months, and finally died of septic shock in August 2013.

## Summary of Other Cases

Three other cases with similar brain MRI findings were identified retrospectively in our hospital (Table 1). The age of the three cases ranged from 41 to 81 years, and included two females and one male. The brief histories of these three cases are described below.

**Table 1:** Clinical characteristics of the cases in the literature and our case series.

Reference	Age (year)/sex	Cancer type	EGFR mutation	Metastasis	Chemotherapy agents used before the brain lesion was diagnosed	Neurological signs	CSF cytology	Paraneoplastic antibodies	Treatments for the brain lesions	Prognosis
Khil et al. (3)	75/M	Lung adenocarcinoma	NA, but supposed to be positive^c^	Bone	Gefitinib	Headache and dizziness	Malignant cells	Negative antibodies in serum	NA	Expired due to respiratory failure 1 month later
Khil et al. (3)	47/F	Lung adenocarcinoma	NA	Brain^b^	Docetaxel and carboplatin	General weakness, seizure, and drowsiness	NA	NA	Whole brain radiotherapy	Hospice care
Crombe et al. (4)	56/M	Lung adenocarcinoma	Positive	Bone, lung	Gefitinib	Diplopia, ataxia, drowsiness, and facial hypoesthesia	Malignant cells	Negative antibodies in serum and CSF	NA	Expired due to respiratory failure 2 months later
Case 1	39/M	Lung adenocarcinoma	Positive, exon 19 deletion	Lung	Vinorelbine, cisplatin, and gefitinib	Dizziness, tinnitus, hearing impairment, seizure, nystagmus, diplopia, and ataxia	Malignant cells	Positive anti-Ma2 antibodies in serum	Intravenous steroid, intravenous immunoglobulin, intrathecal chemotherapy, and Erlotinib	Expired due to septic shock 1 year later
Case 2	Not be shown^a^	Lung adenocarcinoma	Positive	Lung, brain^b^	Vinorelbine, cisplatin, docetaxel, gemcitabine, and gefitinib	Dizziness, unsteady gait, and seizure	NA	NA	Brain radiotherapy	Lost to follow-up
Case 3	Not be shown^a^	Lung adenosquamous carcinoma	Positive, exon 21 L858R point mutation	Lung, bone	No	Dysphagia, dysarthria, general weakness, consciousness changes, and seizure	Negative	Negative antibodies in serum and CSF	Gefitinib, Afatinib	Expired due to respiratory failure 7 months later
Case 4	Not be shown^a^	Lung adenocarcinoma	Positive, exon 19 deletion and codon 790 mutation	Lung, bone, brain^b^	Erlotinib, vinorelbine, pemetrexed, and gemcitabine	Dizziness, unsteady gait, and general weakness	NA	NA	Brain radiotherapy, Osimertinib	Survived over 6 months

*^a^This information cannot be shown because we did not get informed consent from these patients*.

*^b^Brain parenchymal metastasis*.

*^c^The author did not mention if there was EGFR mutation, but the patient received Gefitinib*.

*CSF, cerebrospinal fluid; EGFR, epidermal growth factor receptor; NA, not available*.

**Figure 2:**
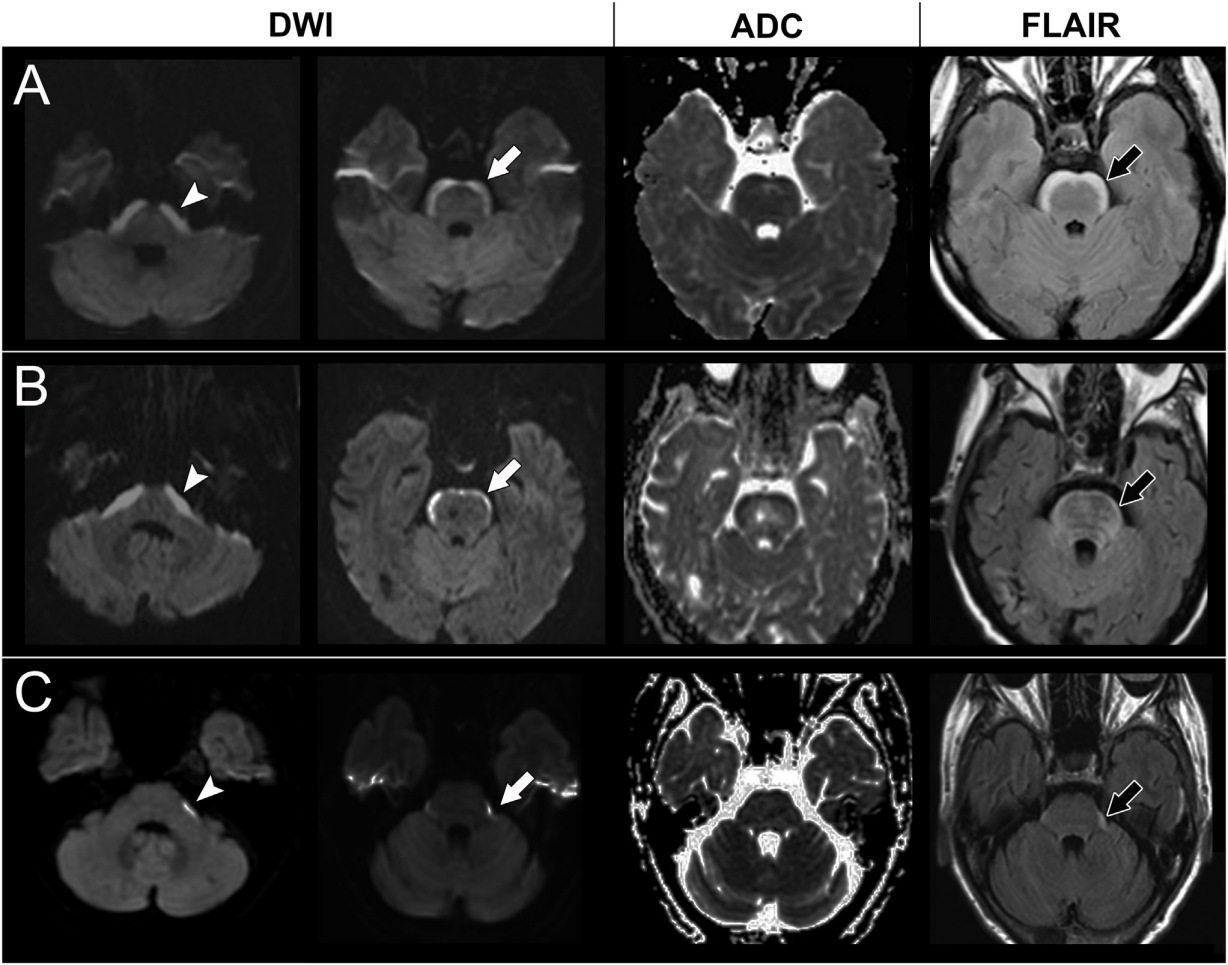
Brain magnetic resonance imaging of case 2, 3, and 4. A high signal in diffusion-weighted imaging (DWI) at the lower pons level (arrow head) and upper pons level (white arrow) with a corresponding low signal on apparent diffusion coefficient (ADC) mapping and hyperintensities on fluid attenuation inversion recovery (FLAIR) imaging (black arrow) in case 2 **(A)**, case 3 **(B)**, and case 4 **(C)**.

The first case had lung adenocarcinoma with EGFR gene mutations and lung-to-lung metastasis. The case received tumor resection, adjuvant chemotherapy (gemcitabine, cisplatin, docetaxel, and vinorelbine), and gefitinib. Four years later, the case presented with progressive dizziness, unsteady gait, and seizures. Brain MRI showed parenchymal metastasis at the left cerebellum and a non-enhanced symmetric band-like anterior brainstem lesion with a restricted diffusion pattern (Figure 2). The case received brain radiotherapy for a short period but was later lost to follow-up.

The second case had progressively slurred speech, swallowing difficulties, and general weakness for 2 weeks before visiting our neurology clinic. Brain MRI showed non-enhanced symmetric band-like anterior brainstem lesions with restricted diffusion (Figure 2). A CSF study showed no pleocytosis but an elevated protein level (119 mg/dl). There were no malignant cells in the CSF cytology. A chest X-ray and chest computed tomography showed lung tumors, and pathology showed lung adenosquamous carcinoma with EGFR gene mutations. Lung-to-lung and bone metastases were also noted. A paraneoplastic antibody survey in serum and CSF were negative. The case received gefitinib treatment; however, the dysphagia and dysarthria progressed further. Follow-up brain MRI showed progressive anterior brainstem lesions, so the physician shifted gefitinib to afatinib for better central nervous system penetration. However, the case became comatose and died 7 months later.

The third case had lung adenocarcinoma with EGFR gene mutations and lung-to-lung and bone metastases. The case received erlotinib, vinorelbine, pemetrexed, and gemcitabine, but the lung cancer still progressed. Two years later, the case suffered from poor appetite, dizziness, general weakness, and unsteady gait. Brain MRI showed numerous enhancing nodules in the bilateral cerebellum and cerebrum, suggesting brain metastasis. The case received whole brain radiotherapy and osimertinib. Follow-up brain MRI showed the disappearance of almost all of the enhancing nodules, but also revealed newly developed restricted diffusion lesions at the anterior brainstem and bilateral corona radiata (Figure 2). However, the clinical condition and lung lesions were stable. The case was kept on osimertinib treatment and was still alive at the time this manuscript was written.

## Literature Review

We reviewed the literature and identified three more cases with similar brain MRI findings (3, 4). The clinical characteristics of the seven cases are summarized in Table 1. There were four males and three females, with an average age of 57 years (range 39–81 years). All of the cases had NSCLC, with six having adenocarcinoma and one adenosquamous carcinoma. All had bone, lung, or brain parenchymal metastasis. Five cases had confirmed EGFR gene mutations. The interval between the diagnosis of lung cancer and discovery of the brainstem lesions ranged from 3 months to 4 years. In one patient (case 3), anterior brainstem lesions led to the diagnosis of lung cancer. Five cases had used a thyroxine kinase inhibitor (TKI) before discovery of the brainstem lesions. The associated neurological symptoms included dizziness, unsteady gait, seizures, diplopia, drowsiness, headache, general weakness, facial hypoesthesia, tinnitus, hearing impairment, dysarthria, and dysphagia. Three cases had malignant cells in CSF, and three other cases had brain parenchymal metastasis. One case had positive anti-Ma2 antibodies in their serum. The brain lesions were treated with radiotherapy in three cases, intrathecal chemotherapy in one case, and TKIs in three cases. The case with positive anti-Ma2 antibodies was treated with intravenous steroids and intravenous immunoglobulin; however, the response was poor. The prognosis seemed to be poor in most of the cases, with survival of less than 1 year after identification of the brain lesions.

## Discussion

The most prominent feature of the anterior brainstem lesions on brain MRI in these patients was a high signal on DWI and FLAIR imaging with a corresponding low signal on ADC mapping. This can be described as a “restricted diffusion” pattern, which is thought to represent cytotoxic edema (5). Restricted diffusion develops most commonly in acute ischemic stroke and sometimes in prolonged status epilepticus, mitochondrial disease, or prion disease (5). However, these etiologies were very unlikely in our patients given their clinical presentations and CSF findings. Restricted diffusion has also been reported in high cellular brain tumors such as lymphoma, high-grade glioma, central necrosis of brain abscesses, and some metastases (6). However, these brain tumors often have strong contrast enhancement on brain MRI (6), which was not found in our cases. The CSF cytology and cultures in our cases also did not favor these etiologies.

Considering that all of the patients had lung cancer, other etiologies such as LC, paraneoplastic neurological syndrome (PNS) or chemotherapy-related toxic encephalopathy may explain the brain lesions. Chemotherapy-related toxic encephalopathy was unlikely because different chemotherapy agents or TKIs were given in six cases, and one case was treatment-naive. PNS was also less likely because the brainstem findings did not fit any known PNS. Moreover, only one case had positive anti-Ma2 antibodies, and this case exhibited a poor response to intravenous steroid and intravenous immunoglobulin treatment. Considering that three cases had malignant cells in their CSF and another three cases had brain parenchymal metastasis, LC was the most likely etiology.

The most common brain MRI finding of LC is diffuse leptomeningeal enhancement (2). However, the brain MRI lesions in our patients were non-enhanced. In fact, restricted diffusion has rarely been reported as an imaging finding for LC. Hu et al. reported a 77-year-old female with lung adenocarcinoma who presented with rapidly progressive consciousness changes (7). Her brain MRI showed restricted diffusion along the surface of the bilateral occipital cortex, and since CSF cytology showed malignant cells, LC was suspected. Ayzenberg et al. also reported a 77-year-old male with lung adenocarcinoma who presented with consciousness changes (8). His brain MRI showed restricted diffusion over bilateral cortical and subcortical areas. He fell into a coma and died 5 days later. A postmortem neuropathological examination was consistent with LC, showing extensive tumor cell infiltration as well as microinfarctions due to intracapillary infiltration of tumor cells *via* Virchow-Robin spaces. We speculate that the anterior brainstem restricted diffusion in our case series may have been caused by malignant cells concentrating in the CSF cistern around the brainstem and then infiltrating into the circumferential perforating arteries along the anterior brainstem surface, thereby resulting in microinfarctions.

Most cases in our series had NSCLC with EGFR mutations, for which survival has been shown to be longer if treated with TKIs; however, the central nervous system was a frequent site of recurrence in these patients (9). The reported median survival after a diagnosis of LC is only a few months (1, 2). In this study, three cases survived for only a few months, two cases had an unknown survival length, one case who received TKIs and intrathecal chemotherapy survived for 1 year, and the other case who received TKIs and brain radiotherapy had survived for more than 6 months when this manuscript was written. Several studies have reported that TKIs with better blood–brain barrier penetration, a higher dosage of TKIs, intrathecal chemotherapy, whole or focal brain radiotherapy, or a combination of multiple treatment strategies may improve the outcome of patients with LC (1, 10).

In conclusion, we reported a novel brain MRI finding with anterior brainstem restricted diffusion in patients with NSCLC. We believe that this is a new imaging finding of LC, and that it may be caused by high cellularity of the tumor cells on the brainstem or microinfarctions due to intracapillary tumor cell infiltration. Because brain MRI is an easily accessible and non-invasive diagnostic tool for LC, the identification of this unique imaging finding could be helpful in diagnosing LC. More case series are warranted and may provide further information about the risk factors and prognosis of patients with this imaging finding. Further studies with a larger number of cases and biopsy or autopsy findings may be able to elucidate the pathological features associated with this imaging finding and support our hypothesis.

## Ethics Statement

This study was approved by the Institutional Review Board of Chang Gung Memorial Hospital. Written informed consent for case 1 has been obtained.

## Author Contributions

C-YC: literature review, manuscript writing, figures making. C-YH: table making, literature review, manuscript writing. Y-HT and S-CC: imaging analysis. K-LL: paraneoplastic antibodies testing. C-EH and Y-HF: cases review. Y-CH: figures making, final manuscript review, and editing.

## Conflict of Interest Statement

The authors declare that the research was conducted in the absence of any commercial or financial relationships that could be construed as a potential conflict of interest.
